# Supplementary figures and images for: Enablement of nursing home residents in infection prevention during general practitioner visits: A qualitative study
Source: PLoS One. 2022 Apr 7;17(4):e0266502. doi: 10.1371/journal.pone.0266502 (PMC8989284; doi:10.1371/journal.pone.0266502)

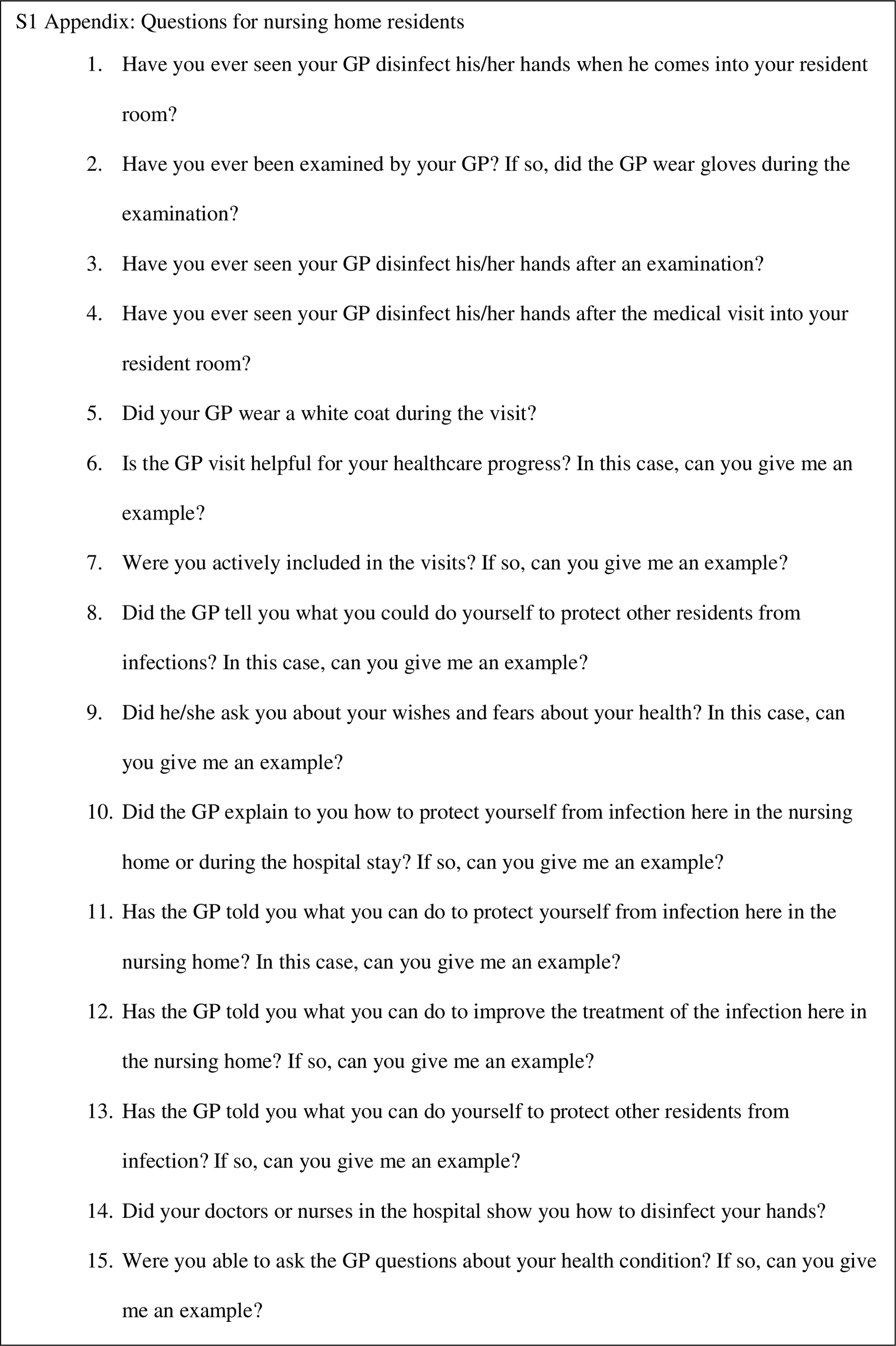

Supplement: S1 Appendix — (TIF) [file pone.0266502.s001.tif]

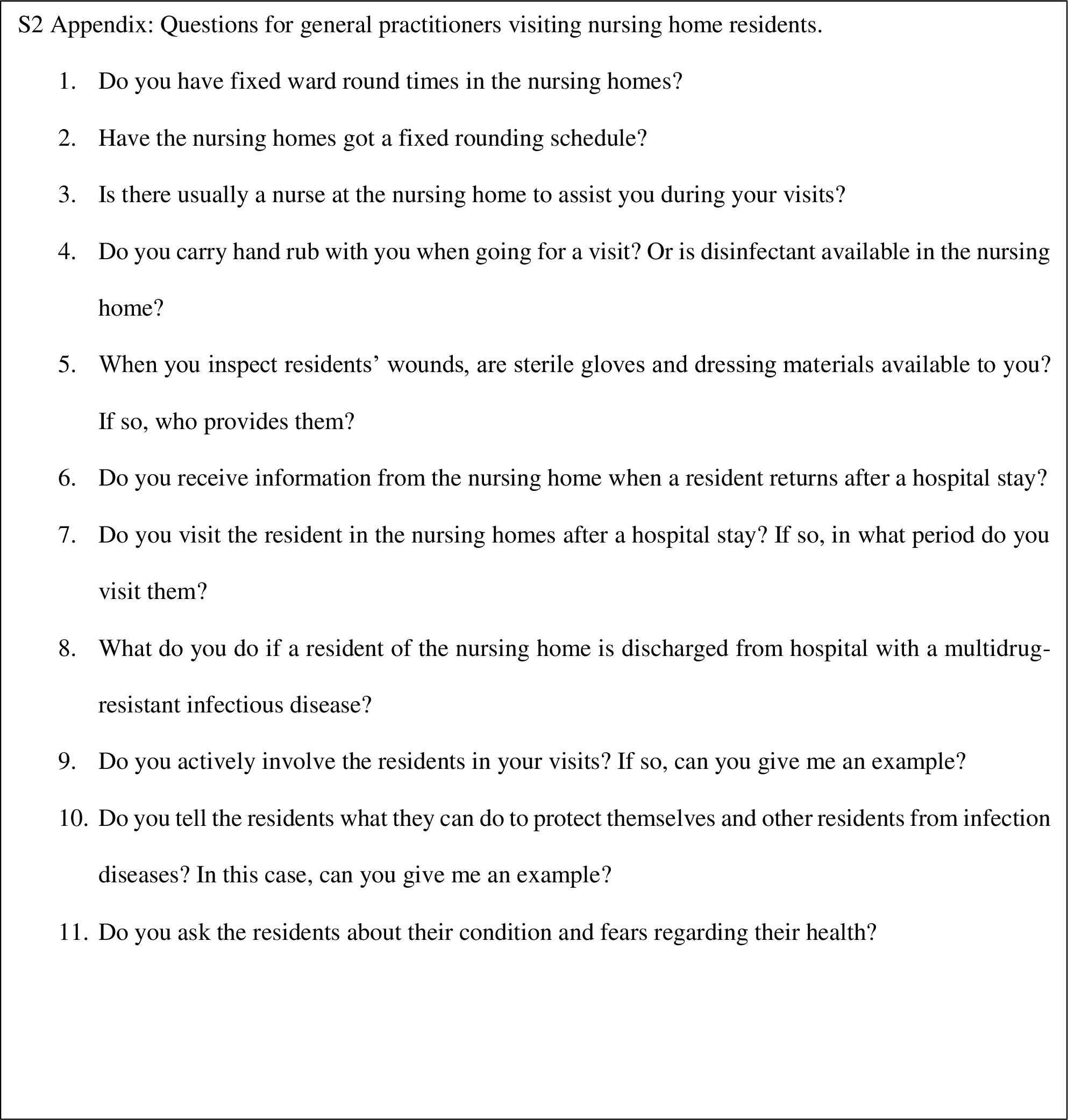

Supplement: S2 Appendix — (TIF) [file pone.0266502.s002.tif]
