# Supplementary material for: Enablement of nursing home residents in infection prevention during general practitioner visits: A qualitative study
Source: PLoS One. 2022 Apr 7;17(4):e0266502. doi: 10.1371/journal.pone.0266502 (PMC8989284; doi:10.1371/journal.pone.0266502)
Supplement: Attachment — Submitted filename: Letter to the Reviewer and Editor_Plos-One.docx [file pone.0266502.s004.docx]

Universitätsklinikum Bonn, Venusberg-Campus 1, 53127 Bonn

**PONE-D-20-34748**

**'Response to Reviewers'**

**Revision: Enablement of nursing home residents in infection prevention during GPs visits: A qualitative study**

16. September 2021

Dear Academic Editor,

Dear Reviewer,

Thank you very much for taking the time and effort to read our manuscript. We appreciate your careful review and valuable suggestions. A revision of the paper has been carried out to take all of them into account. To better understand our modifications, we have highlighted them in yellow and provide an exact line number where we made the changes. Thank you very much for the helpful language corrections. We have implemented them all precisely in the manuscript. We have marked this with "implemented" in the following table.

We have had the entire document professionally proofread.

Kind regards,

The Authors

| **Comment** | **Response** |
| --- | --- |
| **Abstract**  Line 19: General practitioners avoid nosocomial infections' makes it sound like the GP is avoiding getting an infection, rather than the residents. Consider changing to 'prevent nosocomial infections. | Thank you very much for this hint. We revised this. Please see lines: 20-21. |
| Line 31: ´enablement' instead of 'enabling' | Implemented. Please see line 33. |
| Line 33: during the visit to the GP'. Please clarify that the GP visited the resident rather than the other way round. | Implemented. Please see line 36. |
| Line 34: Conclusion: the first sentence doesn't make sense. Please consider rephrasing e.g., 'lack of ability .is due to', or 'For residents to maintain adequate hygiene requires. | Implemented. Please see lines 37 to 40. |
| Line 35: Please consider rephrasing the following sentence to ensure clarity: 'The nursing home residents have a different need to be enabled. | Implemented. Please see lines 37 to 40. |
| **Background**  Line 49 'resistant' rather than 'resistance' | Implemented. Please see line 53. |
| Line 50 Please write 'staph' in full | Implemented. Please see lines 53 and 54. |
| Line 52: bloodstream infections and pneumonia | Implemented. Please see line 57. |
| Line 54: Besides – not quite the right word in this case. Please consider another word. | Removed. |
| Line 56: specify EU member states | We specify 27 European Member States. Please see line 51. |
| Line 56: remove to implement strategies (duplicated) | Removed. |
| Line 55-68: Consider making it into 2 sentences as rather long and poor punctuation. | Implemented. Please see lines 60 to 64. |
| Line 59 – remove the comma after 2017 | Implemented but readded by the professional proofreader. |
| Line 63: Is hand rub better than washing hands with soap and water in this instance, or is it just the convenience of hand rub? It would be interesting to mention this here. | Thank you very much for this important suggestion. We have described it in a more detailed manner. Please see lines 69 to 73. |
| Line 72: GP (rather than GPs) | Removed. |
| Line 73: Please consider clarifying sentence – 'There is not GP with an overview… | Implemented. Please see lines 80 to 81. |
| Line 79: Would be helpful to mention the guidelines you refer to in the Discussion | Thank you very much for this suggestion. We have discussed it in a more detailed manner. Please see part "Discussion". |
| **Recruitment and informed consent**  121: Please give details about how residents gave their consent, to whom, and how it was recorded. | We described it in a more detailed manner. Please see lines 122 to 126. |
| Data collection Please consider adding what language the data was collected in. | Implemented. Please see line 148. |
| Did the researchers have any relationship with the interviewees prior to the study? | We described it in a more detailed manner. Please see lines 134 to 136. |
| 130. Please specify here if the interviews were audio-recorded. | Added. Please see line 148. |
| 131: Please consider the following revision: prevention measures during GP visits, instead of while | Implemented. Please see line 162. |
| 131: Was an interview guide used? Please provide example questions. | Implemented. See lines 140 to 141.  We also added example questions. Please see Box 1 and 2 in the Supplements lines 674 ff. |
| Did you pilot the interview guide before the interviews? | We described it in a more detailed manner. Please see lines 145 to 146. |
| 141: Please consider removing 'and therapies' or rewording the sentence. | Removed. |
| **Data analysis**  Please give details about how were the field notes from the resident interviews were used in the analysis | Thank you for your note. We described it in a more detailed manner. Please see lines 179 to 185. |
| Please give details of how and when the data was translated to English. | Please see lines 156 to 158. |
| 149: Please specify who did the analysis (initials). Were they the same researchers who conducted the interviews? | Thank you for your question. We described it in a more detailed manner. Please see line 166. |
| 151: It is not clear how the five major categories informed the themes described in the findings section. Please give more details about how you arrived at the final themes. | Thank you for your comment. We described this part in a more detailed manner.  Please see lines 147 to 156. |
| Please give details about consideration of data saturation. | We described it in a more detailed manner.  Please see lines 151 to 153. |
| **Results**  It would be helpful to orient the reader by listing the main themes first. | Thank you, that is an excellent suggestion. We listed the five main themes.  Please see lines 186 to 194. |
| Please give some context to the quotations used (e.g., Female GP with *years experience) | Thank you for the excellent comment. We now give additional pieces of information in each interview quotations about the GPs.  Please see the interview quotations in lines 227 ff. |
| 172: Six main categories are now mentioned. Should there be themes? Also, there are only 5. | Thank you and sorry. It was a spelling mistake. There are five main categories.  Please see lines 186 to 194. |
| 176: Theme: this provides an interesting context for the research but seems to move away from the point. I would consider shortening | Thank you very much. We decided not to shorten. The medical visit is the basis where the GP and the resident meet regularly. While visits, the GP should follow all hygiene guidelines, inform the patients, and could form the basis for treating infections, counseling and educating patients about their health literacy in hygiene. |
| 179: 'In case of questions – please give details about what sort of questions are being referred to. Questions from residents or questions from the GP? | Sorry for that confusion; the GPs questions are meant. Please see the line 217. |
| 201: This paragraph is talking about GPs views and behaviors to MRSA and should possibly be discussed within another theme. | We improved the discussion part with this theme. Please see lines 308 to 390. |
| 221: Quotation – it is unclear if this is a single conversation. Also, the full conversation is not necessary to exemplify the findings. Consider shortening to make it more succinct. | Thank you very much for pointing out this unclear description. From our point of view, the external visit by GPs from their office to the nursing home is a critical step in infection prevention. If they do not apply hand hygiene, they could spread germs into nursing homes. We have tried to describe this step more clearly.  Please see lines 248 to 251, 254 to 257, and also 263 to 265. |
| 233: Please make clear what is meant by 'rejecting microbiological differentiation.' | We described it in a more detailed manner. Please see lines 275 to 283. |
| 235: 'without thinking about the effects of all residents' – is this the authors' interpretation of the GPs actions, or was there more information than was given in the quotation? | Thank you very much for this critical detail. We try to describe it now without an interpretation. Please see lines 280 to 283. |
| 237: Please consider truncating the quotation at line 240. The quotation after this point does not add anything further. | We try to describe the result more specific and shorten the quotation. Please see lines 267 to 273. |
| 253: Please consider using another word than 'overwhelmed', which implies that the participants were not able to give an answer to the question. | We implemented the words "try to be polite."  Please see line 301. |
| 271 and 272 – please clarify why some words are in quotation marks. If they are quotations from interviews, please put them in italics. | We put the words in italics. |
| 282: This quotation can also be truncated as the rest talks about how often the GP visits rather than the relationship | We shorten the quotation. Please see lines 334 to 335. |
| 290: Consider rephrasing as follows: None of the residents described receiving hand hygiene training. Please consider adding details about why residents thought this to be the case. | Please see lines 319 to 325, lines 337 to 341, and 348 to 352. |
| 308: Please consider using another term than 'dared' as this word suggests a high element of risk. Residents didn't feel able to talk to their GP? | Implemented. Please see line 364. |
| 317: 'GPs did not discuss infection prevention' – please clarify the sentence as GPs did discuss prevention in the quotation below. | The authors have discussed the sections where the treatment of wounds with sterile dressings and sterile gloves is described by the GPs. We have decided to cut this part of the results as it requires too much detailed knowledge to understand these statements and does not improve the manuscript accordingly. |
| **Discussion**  In general, I felt that the discussion section was not oriented around the main aims of the research: hand hygiene and patient enablement. You could consider structuring your discussion section around TPB. Further discussions on enablement would be helpful.  334: it is not clear how TPB was used in this study. Please give further details. | We thank you for this feedback. We have revised many parts of the Discussion based on your comments. As psychologists and nurse scientists, the TPB guided us as theoretical background in deriving our research question and understanding individual factors that might influence GPs' and residents' hand hygiene behavior. However, we looked at both individual and organizational factors facilitating or hindering adequate hand hygiene behavior. Hence, the theory does not help the reader in understanding our final results and categories. Your comment indicates possible confusion when highlighting the TPB as theoretical background. Thus, we decided to waive the description of the theory to avoid irritation. |
| 338 – 348: This paragraph discusses the context of GP visits to nursing homes, but it is not particularly relevant to the aims of the research. Please consider revising and linking these points to hand hygiene and whether you found any relationship with the types of visits and use of hand hygiene measures. | Thank you for this helpful hint. We have revised this section and elaborated on the GPs' perceived behavior on hand hygiene and the relation to different types of visits.  Please see lines 380 ff. |
| 350-355: Please give some consideration about how the patient-practitioner relationship affects hand hygiene measures | Thank you very much for your comment. We consider this later in the manuscript.  Please see lines 444 to 456. |
| 355: Please comment about whether GPs were asked about their knowledge of hand hygiene regulations. | We described it now in the lines 380 to 390. |
| 365: Discussion about previous antibiotics was not presented in the results section. Is this a new finding? | Thank you for this hint. The sentence is removed. |
| 367: Please consider another term for 'hardly perceived.' | Changed. |
| 367: Please clarify who 'They' are (GPs, residents, or both) | Implemented. "They" refers to "The residents". |
| 375: Remove 'hand.' | Removed. |
| **Limitations**  Did the authors consider interviewing the nursing home staff for their perspectives of hand hygiene measures in nursing homes? | The perspectives, perceived behavior, knowledge, and attitudes of hand hygiene measures from nurses and nursing home managers in the PräNosInAA study was published before (45)  Please see line 477. |
| 382: while results couldn't be generalized to residents with cognitive impairment, there is mention in the findings that a large majority of residents do have dementia. I think it would be helpful to acknowledge this in the Discussion too – and suggest what might be transferable and what could be done in these cases. | Thank you for this very significant comment.  We try to improve this part of the limitations and suggest what is helpful for residents with cognitive impairments.  Please see lines: 470 to 475. |
